# 4-({[4-Amino-6-(*p*-bromo­benz­yl)-5-oxo-4,5-dihydro-1,2,4-triazin-3-yl]sulfan­yl}acet­yl)-3-phenyl­sydnone

**DOI:** 10.1107/S1600536811010798

**Published:** 2011-03-31

**Authors:** Hoong-Kun Fun, Ching Kheng Quah, Nithinchandra, Balakrishna Kalluraya

**Affiliations:** a X-ray Crystallography Unit, School of Physics, Universiti Sains Malaysia, 11800 USM, Penang, Malaysia; b Department of Studies in Chemistry, Mangalore University, Mangalagangotri, Mangalore 574 199, India

## Abstract

In the title compound, C_20_H_15_BrN_6_O_4_S [symstematic name: 4-({[4-amino-6-(*p*-bromo­benz­yl)-5-oxo-4,5-dihydro-1,2,4-triazin-3-yl]sulfan­yl}acet­yl)-3-phenyl-1,2,3-oxadiazol-3-ium-5-olate], the 4,5-dihydro-1,2,4-triazine ring is essentially planar [maximum deviation = 0.020 (1) Å] and is inclined at dihedral angles of 89.06 (9), 82.21 (8) and 83.98 (8)° with respect to the oxadiazol-3-ium, phenyl and benzene rings. The oxadiazol-3-ium ring forms dihedral angles of 52.71 (9) and 8.77 (9)°, respectively, with the phenyl and benzene rings. In the crystal, the mol­ecules are linked *via* pairs of inter­molecular N—H⋯O hydrogen bonds, generating *R*_2_^2^(10) ring motifs and are further linked *via* inter­molecular N—H⋯N and weak C—H⋯O hydrogen bonds into infinite columns along [100].

## Related literature

For general background to and the biological activity of sydnone derivatives, see: Rai *et al.* (2008) ▶; Jyothi *et al.* (2008 ▶). For standard bond-length data, see: Allen *et al.* (1987 ▶). For hydrogen-bond motifs, see: Bernstein *et al.* (1995 ▶). For the stability of the temperature controller used in the data collection, see: Cosier & Glazer (1986 ▶).
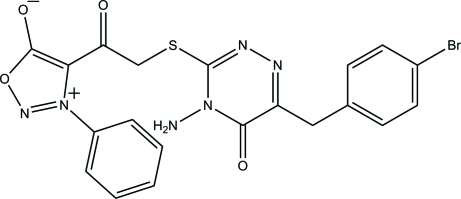


## Experimental

### 

#### Crystal data

C_20_H_15_BrN_6_O_4_S
*M* *_r_* = 515.35
Triclinic, 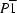

*a* = 6.3842 (3) Å
*b* = 10.0832 (5) Å
*c* = 17.1563 (8) Å
α = 104.873 (1)°
β = 93.507 (1)°
γ = 98.189 (1)°
*V* = 1050.99 (9) Å^3^
*Z* = 2
Mo *K*α radiation
μ = 2.10 mm^−1^
*T* = 100 K
0.32 × 0.26 × 0.06 mm

#### Data collection

Bruker SMART APEXII DUO CCD area-detector diffractometer
Absorption correction: multi-scan (*SADABS*; Bruker, 2009 ▶) *T*_min_ = 0.553, *T*_max_ = 0.892
21938 measured reflections
6161 independent reflections
5241 reflections with *I* > 2σ(*I*)
*R*_int_ = 0.029

#### Refinement

*R*[*F* ^2^ > 2σ(*F* ^2^)] = 0.032
*wR*(*F* ^2^) = 0.089
*S* = 1.03
6161 reflections
297 parameters
H atoms treated by a mixture of independent and constrained refinement
Δρ_max_ = 0.95 e Å^−3^
Δρ_min_ = −0.50 e Å^−3^

### 

Data collection: *APEX2* (Bruker, 2009 ▶); cell refinement: *SAINT* (Bruker, 2009 ▶); data reduction: *SAINT*; program(s) used to solve structure: *SHELXTL* (Sheldrick, 2008 ▶); program(s) used to refine structure: *SHELXTL*; molecular graphics: *SHELXTL*; software used to prepare material for publication: *SHELXTL* and *PLATON* (Spek, 2009 ▶).

## Supplementary Material

Crystal structure: contains datablocks global, I. DOI: 10.1107/S1600536811010798/lh5224sup1.cif

Structure factors: contains datablocks I. DOI: 10.1107/S1600536811010798/lh5224Isup2.hkl

Additional supplementary materials:  crystallographic information; 3D view; checkCIF report

## Figures and Tables

**Table 1:** Hydrogen-bond geometry (Å, °)

*D*—H⋯*A*	*D*—H	H⋯*A*	*D*⋯*A*	*D*—H⋯*A*
N6—H1*N*6⋯N3^i^	0.81 (3)	2.47 (3)	2.9835 (19)	123 (2)
N6—H1*N*6⋯N4^i^	0.81 (3)	2.40 (3)	3.050 (2)	138 (3)
N6—H2*N*6⋯O4^ii^	0.86 (3)	2.15 (3)	2.989 (2)	164 (2)
C14—H14*B*⋯O3^iii^	0.97	2.50	3.416 (2)	157

Symmetry codes: (i) 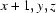; (ii) 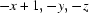; (iii) 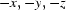.

**Fig. 1.**
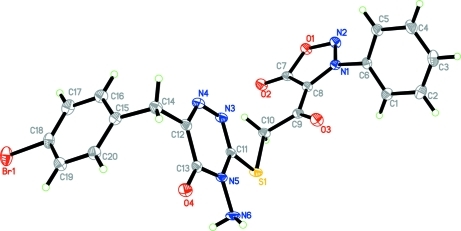
The molecular structure of the title compound showing 50% probability displacement ellipsoids for non-H atoms.

**Fig. 2.**
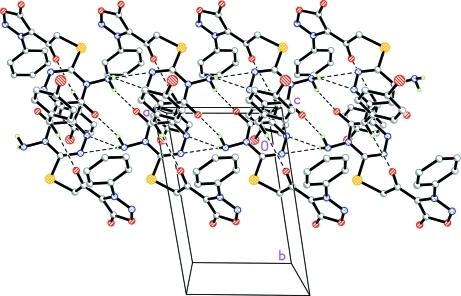
Part of the crystal structure of the title compound, viewed along the c axis. H atoms not involved in hydrogen bonds (dashed lines) have been omitted for clarity.

## supplementary crystallographic information

### Comment

Sydnones are mesoionic heterocyclic aromatic chemical compounds. The study of sydnones remains as a field of interest because of their electronic structures and varied types of biological activities (Rai *et al.*, 2008). Recently sydnone derivatives were found to exhibit promising antimicrobial properties (Jyothi *et al.*, 2008). Since their discovery, sydnones have shown diverse biological activities and it is thought that the mesoionic nature of the sydnone ring promotes significant interactions with biological systems. Photochemical bromination of 3-aryl-4-acetylsydnone affords 3-aryl-4 bromoacetylsydnones. Condensation of 4-amino-6-(4-bromobenzyl)-3- sulfanyl-1,2,4-triazin-5(4H)-one with 3-aryl-4-bromoacetylsydnones yields S-substituted triazinone derivatives (Jyothi *et al.*, 2008).

The molecular structure is shown in Fig. 1. The 4,5-dihydro-1,2,4-triazine ring (N3-N5/C11-C13) is essentially planar [maximum deviation = 0.020 (1) Å at atom N5] and is inclined at angles of 89.06 (9), 82.21 (8) and 83.98 (8) ° with respect to the oxadiazol-3-ium (O1/N1/N2/C7/C8) phenyl (C1-C6) and benzene (C15-C20) rings. The dihedral angles between oxadiazol-3-ium ring (O1/N1/N2/C7/C8) and the phenyl and benzene rings (C1-C6 and C15-C20) are 52.71 (9) and 8.77 (9)°, respectively. The bond lengths (Allen *et al.*, 1987) and angles are within normal ranges.

In the crystal (Fig. 2), the molecules are linked *via* pairs of intermolecular N6–H2N6···O4^ii^ hydrogen bonds (Table 1), generating R_2_^2^(10) ring motifs (Bernstein *et al.*, 1995) and are further linked *via* intermolecular N6–H1N6···N3^i^, N6–H1N6···N4^i^ and weak C14–H14B···O3^iii^ hydrogen bonds (Table 1) into infinite one-dimensional columns along [100].

### Experimental

To a solution of 4-bromoacetyl-3-phenylsydnone (0.01 mol) and 4-amino-6-(4-bromobenzyl)-3-sulfanyl-1,2,4-triazin-5(4H)-one (0.01 mol) in ethanol, catalytic amount of anhydrous sodium acetate was added. The solution was stirred at room temperature for 2 to 3 h. The solid product that separated out was filtered and dried. It was then recrystallized from ethanol. Crystals suitable for X-ray analysis were obtained from 1:2 mixtures of DMF and ethanol by slow evaporation.

### Refinement

H1N6 and H2N6 were located in a difference Fourier map and were refined freely. The remaining H atoms were positioned geometrically and refined using a riding model with C–H = 0.93 or 0.97 Å and *U*_iso_(H) = 1.2 *U*_eq_(C). The highest residual electron density peak is located at 0.88 Å from Br1 and the deepest hole is located at 0.72 Å from Br1.

### Crystal data

**Table:**

C_20_H_15_BrN_6_O_4_S	*Z* = 2
*M**_r_* = 515.35	*F*(000) = 520
Triclinic, *P*1	*D*_x_ = 1.628 Mg m^−^^3^
Hall symbol: -P 1	Mo *K*α radiation, λ = 0.71073 Å
*a* = 6.3842 (3) Å	Cell parameters from 8506 reflections
*b* = 10.0832 (5) Å	θ = 2.5–30.1°
*c* = 17.1563 (8) Å	µ = 2.10 mm^−^^1^
α = 104.873 (1)°	*T* = 100 K
β = 93.507 (1)°	Plate, colourless
γ = 98.189 (1)°	0.32 × 0.26 × 0.06 mm
*V* = 1050.99 (9) Å^3^	

### Data collection

**Table:**

Bruker SMART APEXII DUO CCD area-detector diffractometer	6161 independent reflections
Radiation source: fine-focus sealed tube	5241 reflections with *I* > 2σ(*I*)
graphite	*R*_int_ = 0.029
φ and ω scans	θ_max_ = 30.2°, θ_min_ = 2.1°
Absorption correction: multi-scan (*SADABS*; Bruker, 2009)	*h* = −9→8
*T*_min_ = 0.553, *T*_max_ = 0.892	*k* = −14→14
21938 measured reflections	*l* = −24→24

### Refinement

**Table:**

Refinement on *F*^2^	Primary atom site location: structure-invariant direct methods
Least-squares matrix: full	Secondary atom site location: difference Fourier map
*R*[*F*^2^ > 2σ(*F*^2^)] = 0.032	Hydrogen site location: inferred from neighbouring sites
*wR*(*F*^2^) = 0.089	H atoms treated by a mixture of independent and constrained refinement
*S* = 1.03	*w* = 1/[σ^2^(*F*_o_^2^) + (0.0469*P*)^2^ + 0.5765*P*] where *P* = (*F*_o_^2^ + 2*F*_c_^2^)/3
6161 reflections	(Δ/σ)_max_ = 0.001
297 parameters	Δρ_max_ = 0.95 e Å^−^^3^
0 restraints	Δρ_min_ = −0.50 e Å^−^^3^

### Special details

**Table:**

Experimental. The crystal was placed in the cold stream of an Oxford Cyrosystems Cobra open-flow nitrogen cryostat (Cosier & Glazer, 1986) operating at 100.0 (1) K.
Geometry. All esds (except the esd in the dihedral angle between two l.s. planes) are estimated using the full covariance matrix. The cell esds are taken into account individually in the estimation of esds in distances, angles and torsion angles; correlations between esds in cell parameters are only used when they are defined by crystal symmetry. An approximate (isotropic) treatment of cell esds is used for estimating esds involving l.s. planes.
Refinement. Refinement of F^2^ against ALL reflections. The weighted R-factor wR and goodness of fit S are based on F^2^, conventional R-factors R are based on F, with F set to zero for negative F^2^. The threshold expression of F^2^ > 2sigma(F^2^) is used only for calculating R-factors(gt) etc. and is not relevant to the choice of reflections for refinement. R-factors based on F^2^ are statistically about twice as large as those based on F, and R- factors based on ALL data will be even larger.

### Fractional atomic coordinates and isotropic or equivalent isotropic displacement parameters (Å^2^)

**Table:**

	*x*	*y*	*z*	*U*_iso_*/*U*_eq_	
Br1	0.14494 (4)	0.177407 (19)	−0.446529 (11)	0.02915 (7)	
S1	0.25040 (6)	0.42973 (4)	0.10384 (3)	0.01811 (9)	
O1	−0.50655 (19)	0.68942 (12)	0.23590 (8)	0.0188 (2)	
O2	−0.2801 (2)	0.71471 (13)	0.14077 (8)	0.0208 (3)	
O3	−0.0592 (2)	0.36960 (13)	0.21831 (8)	0.0220 (3)	
O4	0.3090 (2)	−0.01318 (13)	−0.08910 (8)	0.0205 (3)	
N1	−0.3930 (2)	0.52932 (14)	0.27862 (9)	0.0149 (3)	
N2	−0.5387 (2)	0.60958 (15)	0.28897 (9)	0.0186 (3)	
N3	−0.0707 (2)	0.26436 (15)	0.00189 (9)	0.0170 (3)	
N4	−0.1524 (2)	0.14792 (15)	−0.06048 (9)	0.0181 (3)	
N5	0.2602 (2)	0.18642 (14)	0.00230 (9)	0.0143 (3)	
N6	0.4749 (2)	0.22357 (17)	0.03542 (11)	0.0206 (3)	
C1	−0.2106 (3)	0.44195 (17)	0.37978 (11)	0.0196 (3)	
H1A	−0.0839	0.4967	0.3756	0.024*	
C2	−0.2207 (3)	0.36200 (19)	0.43441 (11)	0.0238 (4)	
H2A	−0.0994	0.3628	0.4675	0.029*	
C3	−0.4115 (3)	0.28031 (19)	0.44025 (12)	0.0259 (4)	
H3A	−0.4172	0.2280	0.4777	0.031*	
C4	−0.5930 (3)	0.2768 (2)	0.39032 (12)	0.0248 (4)	
H4A	−0.7196	0.2214	0.3940	0.030*	
C5	−0.5857 (3)	0.35610 (18)	0.33486 (11)	0.0200 (3)	
H5A	−0.7060	0.3541	0.3009	0.024*	
C6	−0.3950 (3)	0.43812 (16)	0.33132 (10)	0.0159 (3)	
C7	−0.3307 (3)	0.65627 (16)	0.19113 (10)	0.0158 (3)	
C8	−0.2595 (3)	0.54977 (16)	0.22188 (10)	0.0147 (3)	
C9	−0.0958 (3)	0.46663 (16)	0.19248 (10)	0.0155 (3)	
C10	0.0227 (3)	0.51392 (17)	0.12772 (11)	0.0171 (3)	
H10A	0.0694	0.6137	0.1461	0.021*	
H10B	−0.0740	0.4948	0.0789	0.021*	
C11	0.1283 (2)	0.28045 (16)	0.02926 (10)	0.0140 (3)	
C12	−0.0334 (3)	0.05691 (17)	−0.09056 (10)	0.0155 (3)	
C13	0.1916 (3)	0.06871 (16)	−0.06161 (10)	0.0150 (3)	
C14	−0.1255 (3)	−0.06046 (17)	−0.16342 (11)	0.0186 (3)	
H14A	−0.2789	−0.0807	−0.1640	0.022*	
H14B	−0.0672	−0.1437	−0.1621	0.022*	
C15	−0.0681 (3)	−0.01499 (16)	−0.23796 (10)	0.0169 (3)	
C16	−0.1898 (3)	0.07165 (18)	−0.26599 (11)	0.0204 (3)	
H16A	−0.3123	0.0922	−0.2421	0.024*	
C17	−0.1300 (3)	0.12721 (18)	−0.32900 (11)	0.0229 (4)	
H17A	−0.2116	0.1841	−0.3479	0.027*	
C18	0.0541 (3)	0.09602 (17)	−0.36308 (11)	0.0204 (3)	
C19	0.1758 (3)	0.00851 (18)	−0.33787 (11)	0.0208 (3)	
H19A	0.2972	−0.0125	−0.3625	0.025*	
C20	0.1132 (3)	−0.04737 (17)	−0.27502 (11)	0.0191 (3)	
H20A	0.1929	−0.1067	−0.2577	0.023*	
H1N6	0.538 (4)	0.226 (3)	−0.0038 (17)	0.032 (7)*	
H2N6	0.512 (4)	0.153 (3)	0.0487 (14)	0.021 (6)*	

### Atomic displacement parameters (Å^2^)

**Table:**

	*U*^11^	*U*^22^	*U*^33^	*U*^12^	*U*^13^	*U*^23^
Br1	0.04790 (14)	0.02057 (9)	0.01944 (10)	0.00057 (8)	0.00410 (8)	0.00878 (7)
S1	0.01188 (18)	0.01887 (18)	0.0231 (2)	0.00468 (14)	0.00303 (15)	0.00339 (15)
O1	0.0194 (6)	0.0176 (5)	0.0236 (6)	0.0084 (4)	0.0060 (5)	0.0094 (5)
O2	0.0218 (6)	0.0192 (6)	0.0270 (7)	0.0074 (5)	0.0063 (5)	0.0132 (5)
O3	0.0233 (6)	0.0210 (6)	0.0275 (7)	0.0115 (5)	0.0070 (5)	0.0118 (5)
O4	0.0215 (6)	0.0212 (6)	0.0228 (6)	0.0122 (5)	0.0050 (5)	0.0075 (5)
N1	0.0147 (6)	0.0137 (6)	0.0175 (7)	0.0047 (5)	0.0021 (5)	0.0048 (5)
N2	0.0192 (7)	0.0186 (6)	0.0221 (7)	0.0086 (5)	0.0057 (6)	0.0088 (5)
N3	0.0129 (6)	0.0191 (6)	0.0196 (7)	0.0047 (5)	0.0035 (5)	0.0046 (5)
N4	0.0138 (6)	0.0205 (6)	0.0205 (7)	0.0030 (5)	0.0039 (5)	0.0060 (5)
N5	0.0105 (6)	0.0176 (6)	0.0183 (7)	0.0064 (5)	0.0035 (5)	0.0080 (5)
N6	0.0103 (6)	0.0258 (7)	0.0270 (8)	0.0081 (5)	0.0019 (6)	0.0064 (6)
C1	0.0211 (8)	0.0175 (7)	0.0207 (8)	0.0038 (6)	0.0011 (6)	0.0057 (6)
C2	0.0283 (9)	0.0234 (8)	0.0210 (8)	0.0065 (7)	−0.0025 (7)	0.0080 (7)
C3	0.0376 (11)	0.0219 (8)	0.0211 (9)	0.0050 (7)	0.0044 (8)	0.0106 (7)
C4	0.0277 (9)	0.0236 (8)	0.0236 (9)	−0.0007 (7)	0.0056 (7)	0.0093 (7)
C5	0.0203 (8)	0.0199 (7)	0.0205 (8)	0.0027 (6)	0.0028 (6)	0.0069 (6)
C6	0.0197 (8)	0.0137 (6)	0.0162 (7)	0.0049 (6)	0.0032 (6)	0.0061 (6)
C7	0.0139 (7)	0.0136 (6)	0.0207 (8)	0.0041 (5)	0.0028 (6)	0.0049 (6)
C8	0.0146 (7)	0.0135 (6)	0.0178 (7)	0.0045 (5)	0.0032 (6)	0.0058 (6)
C9	0.0140 (7)	0.0148 (7)	0.0178 (8)	0.0048 (5)	0.0014 (6)	0.0033 (6)
C10	0.0150 (7)	0.0164 (7)	0.0222 (8)	0.0066 (6)	0.0047 (6)	0.0062 (6)
C11	0.0131 (7)	0.0158 (7)	0.0162 (7)	0.0061 (5)	0.0059 (5)	0.0070 (6)
C12	0.0155 (7)	0.0175 (7)	0.0167 (7)	0.0032 (6)	0.0049 (6)	0.0094 (6)
C13	0.0168 (7)	0.0166 (7)	0.0161 (7)	0.0062 (6)	0.0050 (6)	0.0101 (6)
C14	0.0190 (8)	0.0161 (7)	0.0219 (8)	0.0021 (6)	0.0040 (6)	0.0071 (6)
C15	0.0190 (8)	0.0143 (7)	0.0177 (8)	0.0038 (6)	0.0005 (6)	0.0044 (6)
C16	0.0215 (8)	0.0188 (7)	0.0221 (8)	0.0086 (6)	0.0019 (6)	0.0047 (6)
C17	0.0310 (9)	0.0177 (7)	0.0216 (8)	0.0094 (7)	−0.0017 (7)	0.0062 (6)
C18	0.0301 (9)	0.0150 (7)	0.0158 (8)	0.0018 (6)	0.0018 (7)	0.0047 (6)
C19	0.0219 (8)	0.0216 (8)	0.0201 (8)	0.0059 (6)	0.0055 (7)	0.0056 (6)
C20	0.0217 (8)	0.0176 (7)	0.0203 (8)	0.0078 (6)	0.0021 (6)	0.0068 (6)

### Geometric parameters (Å, °)

**Table:**

Br1—C18	1.9049 (18)	C3—H3A	0.9300
S1—C11	1.7508 (17)	C4—C5	1.390 (3)
S1—C10	1.8020 (16)	C4—H4A	0.9300
O1—N2	1.3680 (19)	C5—C6	1.385 (2)
O1—C7	1.429 (2)	C5—H5A	0.9300
O2—C7	1.200 (2)	C7—C8	1.428 (2)
O3—C9	1.218 (2)	C8—C9	1.465 (2)
O4—C13	1.2187 (19)	C9—C10	1.519 (2)
N1—N2	1.3088 (19)	C10—H10A	0.9700
N1—C8	1.368 (2)	C10—H10B	0.9700
N1—C6	1.445 (2)	C12—C13	1.469 (2)
N3—C11	1.300 (2)	C12—C14	1.504 (2)
N3—N4	1.381 (2)	C14—C15	1.514 (2)
N4—C12	1.300 (2)	C14—H14A	0.9700
N5—C11	1.3680 (19)	C14—H14B	0.9700
N5—C13	1.389 (2)	C15—C20	1.393 (2)
N5—N6	1.4112 (19)	C15—C16	1.401 (2)
N6—H1N6	0.81 (3)	C16—C17	1.389 (3)
N6—H2N6	0.86 (3)	C16—H16A	0.9300
C1—C2	1.383 (3)	C17—C18	1.384 (3)
C1—C6	1.389 (2)	C17—H17A	0.9300
C1—H1A	0.9300	C18—C19	1.386 (2)
C2—C3	1.394 (3)	C19—C20	1.393 (3)
C2—H2A	0.9300	C19—H19A	0.9300
C3—C4	1.389 (3)	C20—H20A	0.9300
			
C11—S1—C10	99.93 (8)	C8—C9—C10	113.52 (13)
N2—O1—C7	110.81 (12)	C9—C10—S1	112.99 (11)
N2—N1—C8	114.46 (14)	C9—C10—H10A	109.0
N2—N1—C6	114.57 (14)	S1—C10—H10A	109.0
C8—N1—C6	130.92 (14)	C9—C10—H10B	109.0
N1—N2—O1	105.56 (13)	S1—C10—H10B	109.0
C11—N3—N4	118.15 (13)	H10A—C10—H10B	107.8
C12—N4—N3	120.73 (14)	N3—C11—N5	124.05 (15)
C11—N5—C13	121.19 (13)	N3—C11—S1	121.47 (12)
C11—N5—N6	116.73 (13)	N5—C11—S1	114.47 (12)
C13—N5—N6	121.61 (13)	N4—C12—C13	123.59 (15)
N5—N6—H1N6	103.2 (19)	N4—C12—C14	118.19 (15)
N5—N6—H2N6	107.7 (15)	C13—C12—C14	118.02 (14)
H1N6—N6—H2N6	103 (2)	O4—C13—N5	122.36 (15)
C2—C1—C6	118.14 (17)	O4—C13—C12	125.46 (16)
C2—C1—H1A	120.9	N5—C13—C12	112.17 (13)
C6—C1—H1A	120.9	C12—C14—C15	107.41 (13)
C1—C2—C3	120.50 (17)	C12—C14—H14A	110.2
C1—C2—H2A	119.8	C15—C14—H14A	110.2
C3—C2—H2A	119.8	C12—C14—H14B	110.2
C4—C3—C2	120.25 (18)	C15—C14—H14B	110.2
C4—C3—H3A	119.9	H14A—C14—H14B	108.5
C2—C3—H3A	119.9	C20—C15—C16	119.23 (16)
C3—C4—C5	120.06 (18)	C20—C15—C14	121.51 (15)
C3—C4—H4A	120.0	C16—C15—C14	119.03 (15)
C5—C4—H4A	120.0	C17—C16—C15	120.87 (17)
C6—C5—C4	118.46 (17)	C17—C16—H16A	119.6
C6—C5—H5A	120.8	C15—C16—H16A	119.6
C4—C5—H5A	120.8	C18—C17—C16	118.47 (16)
C5—C6—C1	122.58 (16)	C18—C17—H17A	120.8
C5—C6—N1	118.14 (15)	C16—C17—H17A	120.8
C1—C6—N1	119.16 (15)	C17—C18—C19	122.11 (17)
O2—C7—C8	136.11 (16)	C17—C18—Br1	119.06 (14)
O2—C7—O1	120.39 (14)	C19—C18—Br1	118.83 (14)
C8—C7—O1	103.49 (14)	C18—C19—C20	118.84 (16)
N1—C8—C7	105.67 (13)	C18—C19—H19A	120.6
N1—C8—C9	126.27 (14)	C20—C19—H19A	120.6
C7—C8—C9	127.67 (15)	C19—C20—C15	120.45 (16)
O3—C9—C8	122.63 (16)	C19—C20—H20A	119.8
O3—C9—C10	123.85 (15)	C15—C20—H20A	119.8
			
C8—N1—N2—O1	0.65 (18)	N4—N3—C11—N5	−2.7 (2)
C6—N1—N2—O1	178.30 (13)	N4—N3—C11—S1	176.05 (12)
C7—O1—N2—N1	−0.32 (17)	C13—N5—C11—N3	4.4 (2)
C11—N3—N4—C12	0.3 (2)	N6—N5—C11—N3	176.67 (16)
C6—C1—C2—C3	−0.1 (3)	C13—N5—C11—S1	−174.36 (12)
C1—C2—C3—C4	0.9 (3)	N6—N5—C11—S1	−2.14 (19)
C2—C3—C4—C5	−0.7 (3)	C10—S1—C11—N3	6.15 (16)
C3—C4—C5—C6	−0.4 (3)	C10—S1—C11—N5	−175.02 (12)
C4—C5—C6—C1	1.3 (3)	N3—N4—C12—C13	0.4 (2)
C4—C5—C6—N1	−174.73 (16)	N3—N4—C12—C14	−174.35 (15)
C2—C1—C6—C5	−1.1 (3)	C11—N5—C13—O4	176.57 (15)
C2—C1—C6—N1	174.91 (15)	N6—N5—C13—O4	4.7 (2)
N2—N1—C6—C5	51.6 (2)	C11—N5—C13—C12	−3.4 (2)
C8—N1—C6—C5	−131.18 (18)	N6—N5—C13—C12	−175.22 (15)
N2—N1—C6—C1	−124.54 (17)	N4—C12—C13—O4	−178.76 (17)
C8—N1—C6—C1	52.6 (2)	C14—C12—C13—O4	−4.1 (2)
N2—O1—C7—O2	179.06 (15)	N4—C12—C13—N5	1.2 (2)
N2—O1—C7—C8	−0.09 (17)	C14—C12—C13—N5	175.89 (14)
N2—N1—C8—C7	−0.71 (19)	N4—C12—C14—C15	92.39 (18)
C6—N1—C8—C7	−177.89 (16)	C13—C12—C14—C15	−82.61 (17)
N2—N1—C8—C9	−173.89 (15)	C12—C14—C15—C20	93.65 (18)
C6—N1—C8—C9	8.9 (3)	C12—C14—C15—C16	−80.78 (19)
O2—C7—C8—N1	−178.5 (2)	C20—C15—C16—C17	−1.1 (3)
O1—C7—C8—N1	0.44 (17)	C14—C15—C16—C17	173.41 (16)
O2—C7—C8—C9	−5.4 (3)	C15—C16—C17—C18	−0.6 (3)
O1—C7—C8—C9	173.50 (15)	C16—C17—C18—C19	1.9 (3)
N1—C8—C9—O3	−1.7 (3)	C16—C17—C18—Br1	−177.44 (13)
C7—C8—C9—O3	−173.40 (16)	C17—C18—C19—C20	−1.4 (3)
N1—C8—C9—C10	178.64 (15)	Br1—C18—C19—C20	177.91 (13)
C7—C8—C9—C10	6.9 (2)	C18—C19—C20—C15	−0.4 (3)
O3—C9—C10—S1	−9.0 (2)	C16—C15—C20—C19	1.6 (3)
C8—C9—C10—S1	170.66 (11)	C14—C15—C20—C19	−172.79 (16)
C11—S1—C10—C9	87.24 (13)		

### Hydrogen-bond geometry (Å, °)

**Table:**

*D*—H···*A*	*D*—H	H···*A*	*D*···*A*	*D*—H···*A*
N6—H1N6···N3^i^	0.81 (3)	2.47 (3)	2.9835 (19)	123 (2)
N6—H1N6···N4^i^	0.81 (3)	2.40 (3)	3.050 (2)	138 (3)
N6—H2N6···O4^ii^	0.86 (3)	2.15 (3)	2.989 (2)	164 (2)
C14—H14B···O3^iii^	0.97	2.50	3.416 (2)	157

Symmetry codes: (i) *x*+1, *y*, *z*; (ii) −*x*+1, −*y*, −*z*; (iii) −*x*, −*y*, −*z*.

## References

[bb1] Allen, F. H., Kennard, O., Watson, D. G., Brammer, L., Orpen, A. G. & Taylor, R. (1987). *J. Chem. Soc. Perkin Trans. 2*, pp. S1–19.

[bb2] Bernstein, J., Davis, R. E., Shimoni, L. & Chang, N.-L. (1995). *Angew. Chem. Int. Ed. Engl.* **34**, 1555–1573.

[bb3] Bruker (2009). *APEX2*, *SAINT* and *SADABS* Bruker AXS Inc., Madison, Wisconsin, USA.

[bb4] Cosier, J. & Glazer, A. M. (1986). *J. Appl. Cryst.* **19**, 105–107.

[bb5] Jyothi, C. H., Girisha, K. S., Adithya, A. & Kalluraya, B. (2008). *Eur. J. Med. Chem.* **43**, 2831–2834. 10.1016/j.ejmech.2008.02.003 18387710

[bb6] Rai, N. S., Kalluraya, B., Lingappa, B., Shenoy, S. & Puranic, V. G. (2008). *Eur. J. Med. Chem*, **43**, 1715–1720. 10.1016/j.ejmech.2007.08.002 17923171

[bb7] Sheldrick, G. M. (2008). *Acta Cryst.* A**64**, 112–122. 10.1107/S0108767307043930 18156677

[bb8] Spek, A. L. (2009). *Acta Cryst.* D**65**, 148–155. 10.1107/S090744490804362X PMC2631630 19171970

